# Early onset frontotemporal dementia with psychiatric presentation due to the C9ORF72 hexanucleotide repeat expansion: a case report

**DOI:** 10.1186/s12883-014-0228-6

**Published:** 2014-11-30

**Authors:** Carla Gramaglia, Roberto Cantello, Emanuela Terazzi, Miryam Carecchio, Sandra D’Alfonso, Nunzia Chieppa, Francesca Ressico, Maria Cristina Rizza, Patrizia Zeppegno

**Affiliations:** Institute of Psychiatry, Dipartimento di Medicina Traslazionale, Università degli Studi del Piemonte Orientale, Novara, Italy; Institute of Neurology, Dipartimento di Medicina Traslazionale, Università degli Studi del Piemonte Orientale, Novara, Italy; SC Neurologia, AOU Maggiore della Carità, Novara, Italy; Dipartimento di Scienze della Salute, Università degli Studi del Piemonte Orientale, Novara, Italy; SC Psichiatria AOU Maggiore della Carità, Novara, Italy

**Keywords:** Frontotemporal dementia, C9Orf72, Psychiatric Presentation, Behavioural variant

## Abstract

**Background:**

Frontotemporal dementia (FTD) may present with psychiatric symptoms, usually together with neurological ones and in cases with a family history of dementia. We describe the case of an FTD behavioural variant with a psychiatric presentation and a normal neurological examination, due to a C9Orf72 gene mutation.

**Case presentation:**

The patient was a 57 years-old Caucasian woman with a recent onset of bizarre behaviours and mystic delusions. She had a negative clinical history for previous psychiatric disorders and treatments and this was her first admission to a Psychiatry Ward. A careful assessment was performed including, beyond psychiatric evaluation, the following: blood sampling, neurological examination (including electroencephalogram, electroencephalogram with zygomatic electrodes, Positron Emission Tomography, Cerebrospinal Fluid Analysis), carotid artery Doppler ultrasound, brain Magnetic Resonance Imaging – angio Magnetic Resonance Imaging. Blood sampling for the genetic assessment of mutations associated to primary dementias was performed as well: the genes investigated were FUS, C9Orf72, PSEN-1, PSEN-2.

**Conclusions:**

Serological tests were negative, neurological examination was normal, instrumental examinations showed theta waves in the posterior temporal areas bilaterally and frontotemporal cortical atrophy bilaterally. The genetic assessment of mutations associated revealed she carried a GGGGCC hexanucleotide repeat expansion (at least 80 repeats) in C9Orf72 intron 1. Patients carrying the C9Orf72 mutation are likely to receive a psychiatric diagnosis (mainly mood disorder or schizophrenia) prior to correct diagnosis; this may be particularly problematic for those patients with no neurological signs to orientate diagnosis. Understanding the manner in which such FTD variant may present as a psychiatric syndrome, with a negative neurological examination, is essential to provide the best treatment for patients, as soon as possible, especially when the behavioural anomalies interfere with their care.

## Background

Frontotemporal dementia (FTD) is one of the most common young-onset dementia and its clinical presentation is characterised by progressive behavioural change, executive dysfunction and language difficulties [1]. The clinical spectrum of FTD encompasses three distinct syndromes: the behavioural variant (bvFTD) and the language variants, semantic dementia (SD) and progressive non-fluent aphasia (PNFA). FTD often overlaps with motor neuron disease (FTD-MND or FTD-ALS), as well as parkinsonian syndromes, progressive supranuclear palsy (PSP) and corticobasal syndrome (CBS) [2]. A positive family history is reported in 30-50% of patients suffering from bvFTD, while patients with SD or PNFA have a much lower frequency [3]. In 2006, a locus on chromosome 9p21 was associated with a large proportion of ALS and FTD cases [4]. Recently, two independent groups reported identification of the FTD/ALS gene defect on chromosome 9p as being a massively expanded GGGGCC hexanucleotide repeat in a non-coding region of the chromosome 9 open reading frame 72 gene (C9Orf72) [5,6]. The hexanucleotide expansion leads to the loss of an alternatively spliced transcript and the accumulation of RNA fragments composed of the repeated nucleotides as RNA foci in the nucleus and/or cytoplasm of affected cells [7]. Such foci may be toxic: they have been shown to sequester some RNA-binding proteins leading to dysregulation of alternative mRNA splicing [8]. Even though further studies are required, aberrant RNA splicing is a highly plausible mechanism in chromosome 9p-linked FTD/ALS given the accumulating evidence for RNA misprocessing in the pathogenesis of both ALS and FTD [9].

The phenotypic spectrum of bvFTD and its pathophysiologic basis have yet to be fully defined. Psychiatric manifestations including delusions, hallucinations, and severe anxiety disorders may be presenting features, as described by Arighi and coworkers [10] in three cases with a positive family history of dementia; only one of the three cases described, anyway, had not neurological symptoms. Although a precise neuroanatomic-phenotypic correlation has yet to be described, some clinical features, in particular neuropsychiatric symptoms, may be due to an altered cortico-thalamo-cerebellar network [11].

## Case presentation

We describe here the case of a 57 years-old woman who came to our attention in October, 2012 because of bizarre behaviours and mystic delusions.

She had completed junior high school, had worked until aged 54 and then retired to look after her grandson.

Her clinical history was negative for previous psychiatric diagnoses and treatment. Her medical history was significant for a cryptogenetic epilepsy: at the time of our examination she was under treatment with Levetiracetam 1000 mg/day. Interestingly, her mother died aged 59, suffering from a rapidly progressive dementia (duration of illness: 11 months); while her father died aged 67 because of myocardial infarction. Her three siblings, aged 45, 47 and 53, were healthy, and so was her offspring, although it cannot be excluded that they develop neurological or psychiatric symptoms in the future.

When admitted to our Day Hospital service, she was also taking Escitalopram as prescribed by her Neurologist who diagnosed an “anxious-depressive syndrome due to worries about her relatives’ health problems”.

On examination, the patient was alert, partially oriented and well groomed, she was overly familiar, perseverative and critical of others (her husband above all) throughout the interview. She showed poor insight into her symptoms, making excuses for her behaviours. She reported episodes of amnesia without consciousness alterations and she complained she could not recognize familiar faces and places, together with insomnia (early awakenings) and coloured visions. During the interviews, a memory impairment emerged together with episodes of confabulation and an inconstant attention.

Her husband described bizarre behaviours, such as childish tantrums, sleeping at eating times in order to avoid having dinner with her husband, complaining about him beating her “because he is a nasty boy”. Moreover, the patient’s religious interests had progressively increased until they severely interfered with daily activities. The patient wouldn’t give explanations for her behaviours and she would only say “it’s my husband’s fault, he always tells me off”.

### Clinical assessment

Blood sampling was performed, and the patient resulted negative for HBV, HCV, HIV and pox.

Her neurological examination was normal except for a facetious attitude. No disorientation, focal signs, altered reflexes nor abnormal plantar responses were detectable. Neither Parkinsonism nor Motor Neuron Disease (MND) signs were observed.

Her clinical assessment also included an elecroencephalogram (EEG), followed by an EEG with zygomatic electrodes which both showed theta waves in the posterior temporal areas, bilaterally.

The patient also underwent carotid artery Doppler ultrasound, which revealed a regular lumen of the common, internal and external carotid and of the vertebral artery, bilaterally.

Her brain MRI – angio MRI showed a marked frontotemporal cortical atrophy, bilaterally, with a broadening of cortical burrows and ventricula. The cerebellum cortex also presented atrophy. The left fronto-opercolar subcortical white matter showed altered signal consistent with previous small ischemic sequelae.

### Psychometric assessment

Her Minnesota Multiphasic Personality Inventory-2 (MMPI-2) was consistent with a tendency to pessimism and difficulties as regards integration in a group. Her relationship with the environment appeared characterized by a tendency to neurotic exploitation, by means of hypochondriac – hysteric symptoms. This means that her relationship with other people was characterized by the unconscious tendency to try to obtain attention and care through her physical symptoms, instead of expressing herself in a clear and assertive way. In stressful situations, she might show affective lability and impulsiveness. She complained feeling hostile and aggressive and she did not express her feelings in an assertive way. She used a “somatic language” in order to express her psychological and emotional issues, while appearing unhappy, confused and uneasy in social relations. Patients with such results are usually diagnosed with a schizotypic personality with a chronic tendency to hypochondriac conversion both of anxiety and hostility feelings, meaning that they usually “replace” the expression of their feelings and emotions (especially anxiety) with physical symptoms and somatic complaints.

At the Wechsler Adult Intelligent Scale- Revised (WAIS-R), she scored as follows: Global IQ = 77; Verbal IQ = 71; Performance IQ = 90. Her performance was particularly poor on Comprehension, Analogies, Arithmetic Reasoning and Stories Rearrangement. Being her Performance IQ 19 points greater than her Verbal IQ, we further investigated her profile, as Literature suggests when scores differ by more than 15 points. A Performance IQ greater than the Verbal IQ suggests a left hemisphere damage. Our patient scored rather low at each of the subtests, particularly we observed: low interest and difficulty retrieving information, scarce fluid intelligence possibly due to brain damage, low motivation, inadequate attention and concentration abilities, difficulties controlling feelings, low thought flexibility, poor ideas, abstraction difficulties, concrete thought.

The patient also underwent a neuropsychological screening test (Milan Overall Dementia Assessment - MODA): her score (82.4) was lower than expected according to age (range: 85.5-89.1). Her performance was worse on “Temporal Orientation”, “Verbal Intelligence” and “Verbal Production”. She was later administered the Frontal Assessment Battery (FAB) in order to globally assess the effectiveness of her executive functions. The score she obtained (14/18) is slightly higher than the cut-off value (12), and indicates poor performances at the “Affinity” and “Phonemic Fluency” trials. Finally, she underwent in-depth neuropsychological tests in order to evaluate her ability to search for words, by means of the “Fluency Test”, and verbal abilities requiring judgment capacities, by means of the “Verbal Judgment” tests. The patient scored as “pathologic” at the items Phonemics and Semantics (both scores = 0). She scored 21/60 at the item “Verbal Judgment”, which is definitely lower than the range values (M = 51, DS = 6.42), thus indicating a deficit of reasoning abilities, conceptualization, categorization and critical-logical abilities.

During her Day Hospital treatment at the Institute of Psychiatry in Novara, Escitalopram was withdrawn and a treatment with Promazine up to 60 mg/day was initiated. Her bizarre behaviours gradually diminished, and she obtained a restful sleep again.

As a consequence of the Neurologic examinations she underwent, the Neurologist suggested further investigation requiring hospitalization at the Neurology ward. She then underwent CSF analysis which revealed normal values of amyloid protein (862, range values >500 pg/ml), tau (147, range values <450 pg/ml), fosfotau (27, range values <61 pg/ml), and plasmatic progranulin (126, range values >61.55 ng/ml).

The brain PET performed with 18FDG was consistent with a cortical non homogenous distribution of the marker, related to the renown atrophy.

Given these results, upon the patient's next of kin (her husband) written informed consent, the patient underwent blood sampling for the genetic assessment of mutations associated to primary dementias: on a first step, the genes investigated were FUS and C9Orf72; DNA was stored for further analyses to be performed including PSEN-1 and PSEN-2 genes, that were not analyzed at first due to the normal CSF profile and the very low mutational frequency in primary dementias. Such assessment revealed she carried a GGGGCC hexanucleotide repeat expansion (at least 80 repeats) in C9Orf72 intron 1. No mutations were found in the FUS gene; PSEN-1 and PSEN-2 were not analyzed.

## Conclusions

Our patient presented a GGGGCC hexanucleotide repeat expansion of C9Orf72: some Authors [5] claim that the GGGGCC repeat length in healthy individuals ranges from 2–23 hexanucleotide units, whereas they estimate the repeat length to be 700–1600 units in FTD/ALS patients based on DNA from lymphoblast cell lines. Yet, accurate sizing of the repeat is challenging, especially in DNA extracted from peripheral blood and brain tissue samples, and the same Authors state that the minimal repeat size needed to cause FTD/ALS still has to be determined and may be significantly smaller.

C9Orf72 neuroimaging assessment is characterized by a greater atrophy in the cerebellum compared to sporadic FTD patients and age-matched healthy controls [12]. Thus, cerebellar atrophy and its concomitant dysfunction might represent a biomarker for a C9Orf72 mutation. However, patients carrying the C9Orf72 mutation do not show classic cerebellar dysfunction symptoms, such as ataxia, and dysarthria. Nonetheless, cerebellar patients are often described as presenting with an intellectual functioning impairment, together with emotional or psychiatric disturbances. Recent evidence suggests that cerebellar patients can show cognitive and affective deficits, in particular executive dysfunction, impaired spatial memory, and personality changes such as disinhibited or inappropriate behaviour, which sometimes raises to psychotic features [13]. Such psychiatric disturbances due to cerebellar dysfunction are in accordance with the finding that C9Orf72 patients show a higher incidence of psychiatric symptoms compared to sporadic FTD cases [14]. As recently reported, psychotic symptoms may be associated with the C9Orf72 repeat expansion, in some cases even in the absence of neurological signs and with no evidence of atrophy on neuroimaging [15]. Understanding the manner in which such FTD variant may present as a neuropsychiatric syndrome is essential to avoid wrong diagnoses and provide the best treatment for patients. Screening for the C9Orf72 repeat expansion may be suggested in patients with late-onset psychotic symptoms, with no previous history of psychiatric disorders [15], who need to be carefully differentiated from subjects affected by schizophrenia, that usually presents at a younger age.

The patient we described actually came to the attention of Psychiatrists earlier than of Neurologists, being her main symptoms represented by bizarre behaviours and mystic delusions, while her memory impairment and executive dysfunction only emerged during our examination. Moreover, her neurologic examination kept normal, faced with imaging evidence of cortical and cerebellar atrophy, similarly to the case described by Arighi and coworkers [10]. Interestingly, the patient's CSF biomarkers were all in normal range, as previously reported in FTD patients carrying GRN [16] and MAPT mutations [17]. In these patients, CSF concentration of tau and P-181 tau protein, classically considered markers of neurodegeneration, was reported to be either normal or borderline, despite extensive neuronal loss observed on brain imaging and severe symptoms. Our patient's CSF findings are therefore in line with previous similar C9Orf72 cases reported.

Our case report is meant to underline the importance of a correct family history collection (our patient’s siblings and offspring should also undergo genetic assessment) and subsequent diagnosis. Even though treatments which have been used in bvFDT to date are all symptomatic, and no specific treatment for psychosis in FTD is currently available [18], a correct diagnosis may allow an improvement of the patients’ quality of life, especially when the behavioral anomalies interfere with their care. That is the case of antipsychotics, such as Promazine, which are particularly effective in case of psychomotor agitation, and proved an effective and tolerable treatment in our patient.

## Consent

Considering the patient's diagnosis, written informed consent for publication of this Case Report and any accompanying images was obtained from the patient's next of kin (her husband). A copy of the written consent is available for review to the Editor of this journal.

## References

[1] Seelaar H, Rohrer JD, Pijnenburg YA, Fox NC, van Swieten JC (2011). Clinical, genetic and pathological heterogeneity of frontotemporal dementia: a review. J Neurol Neurosurg Psychiatry.

[2] Kertesz A, McMonagle P, Blair M, Davidson W, Munoz DG (2005). The evolution and pathology of frontotemporal dementia. Brain.

[3] Seelaar H, Kamphorst W, Rosso SM, Azmani A, Masdjedi R, de Koning I, Maat-Kievit JA, Anar B, Donker Kaat L, Breedveld GJ, Dooijes D, Rozemuller JM, Bronner IF, Rizzu P, van Swieten JC (2008). Distinct genetic forms of frontotemporal dementia. Neurology.

[4] Morita M, Al-Chalabi A, Andersen PM, Hosler B, Sapp P, Englund E, Mitchell JE, Habgood JJ, De Belleroche J, Xi J, Jongjaroenprasert W, Horvitz HR, Gunnarsson LG, Brown RH (2006). A locus on chromosome 9 confers susceptibility to amyotrophic lateral sclerosis and frontotemporal dementia. Neurology.

[5] DeJesus-Hernandez M, Mackenzie IR, Boeve BF, Boxer AL, Baker M, Rutherford NJ, Nicholson AM, Finch NA, Flynn H, Adamson J, Kouri N, Wojtas A, Sengdy P, Hsiung GY, Karydas A, Seeley WW, Josephs KA, Coppola G, Geschwind DH, Wszolek ZK, Feldman H, Knopman DS, Petersen RC, Miller BL, Dickson DW, Boylan KB, Graff-Radford NR, Rademakers R (2011). Expanded GGGGCC hexanucleotide repeat in noncoding region of C9ORF72 causes chromosome 9p-Linked FTD and ALS. Neuron.

[6] Renton AE, Majounie E, Waite A, Simón-Sánchez J, Rollinson S, Gibbs JR, Schymick JC, Laaksovirta H, van Swieten JC, Myllykangas L, Kalimo H, Paetau A, Abramzon Y, Remes AM, Kaganovich A, Scholz SW, Duckworth J, Ding J, Harmer DW, Hernandez DG, Johnson JO, Mok K, Ryten M, Trabzuni D, Guerreiro RJ, Orrell RW, Neal J, Murray A, Pearson J, Jansen IE (2011). A hexanucleotide repeat expansion in C9ORF72 is the cause of chromosome 9p21-linked ALS-FTD. Neuron.

[7] Goedert M, Ghetti B, Spillantini MG (2012). Frontotemporal Dementia: implications for understanding Alzheimer disease. Cold Spring Harb Perspect Med.

[8] Sofola OA, Jin P, Qin Y, Duan R, Liu H, de Haro M, Nelson DL, Botas J (2007). RNA-binding proteins hnRNP A2/B1 and CUGBP1 suppress fragile X CGG premutation repeat-induced neurodegeneration in a Drosophila model of FXTAS. Neuron.

[9] Bäumer D, Ansorge O, Almeida M, Talbot K (2010). The role of RNA processing in the pathogenesis of motor neuron degeneration. Expert Rev Mol Med.

[10] Arighi A, Fumagalli GG, Jacini F, Fenoglio C, Ghezzi L, Pietroboni AM, De Riz M, Serpente M, Ridolfi E, Bonsi R, Bresolin N, Scarpini E, Galimberti D (2012). Early onset behavioral variant frontotemporal dementia due to the C9ORF72 hexanucleotide repeat expansion: psychiatric clinical presentations. J Alzheimers Dis.

[11] Mahoney CJ, Beck J, Rohrer JD, Lashley T, Mok K, Shakespeare T, Yeatman T, Warrington EK, Schott JM, Fox NC, Rossor MN, Hardy J, Collinge J, Revesz T, Mead S, Warren JD (2012). Frontotemporal dementia with the C9ORF72 hexanucleotide repeat expansion: clinical, neuroanatomical and neuropathological features. Brain.

[12] Whitwell JL, Weigand SD, Boeve BF, Senjem ML, Gunter JL, DeJesus-Hernandez M, Rutherford NJ, Baker M, Knopman DS, Wszolek ZK, Parisi JE, Dickson DW, Petersen RC, Rademakers R, Jack CR, Josephs KA (2012). Neuroimaging signatures of frontotemporal dementia genetics: C9ORF72, tau, progranulin and sporadics. Brain.

[13] Schmahmann JD, Caplan D (2006). Cognition, emotion and the cerebellum. Brain.

[14] Snowden JS, Rollinson S, Thompson JC, Harris JM, Stopford CL, Richardson AM, Jones M, Gerhard A, Davidson YS, Robinson A, Gibbons L, Hu Q, DuPlessis D, Neary D, Mann DM, Pickering-Brown SM (2012). Distinct clinical and pathological characteristics of frontotemporal dementia associated with C9ORF72 mutations. Brain.

[15] Galimberti D, Fenoglio C, Serpente M, Villa C, Bonsi R, Arighi A, Fumagalli GG, Del Bo R, Bruni AC, Anfossi M, Clodomiro A, Cupidi C, Nacmias B, Sorbi S, Piaceri I, Bagnoli S, Bessi V, Marcone A, Cerami C, Cappa SF, Filippi M, Agosta F, Magnani G, Comi G, Franceschi M, Rainero I, Giordana MT, Rubino E, Ferrero P, Rogaeva E (2013). Autosomal dominant frontotemporal lobar degeneration due to the C9ORF72 hexanucleotide repeat expansion: late-onset psychotic clinical presentation. Biol Psychiatry.

[16] Carecchio M, Fenoglio C, Cortini F, Comi C, Benussi L, Ghidoni R, Borroni B, De Riz M, Serpente M, Cantoni C, Franceschi M, Albertini V, Monaco F, Rainero I, Binetti G, Padovani A, Bresolin N, Scarpini E, Galimberti D (2011). Cerebrospinal fluid biomarkers in Progranulin mutations carriers. J Alzheimers Dis.

[17] Rosso SM, van Herpen E, Pijnenburg YAL, Schoonenboom NSM, Scheltens P, Heutink P, van Swieten JC (2003). Total tau and phosporylated tau 181 levels in the cerebrospinal fluid of patients with frontotemporal dementia due to P301 and G272 tau mutations. Arch Neurol.

[18] Shinagawa S, Nakajima S, Plitman E, Graff-Guerrero A, Mimura M, Nakayama K, Miller BL: **Psychosis in Frontotemporal Dementia.** *J Alzheimers Dis* 2014, [Epub ahead of print]. 10.3233/JAD-140312 24898651

